# Male partner attendance at antenatal care and adherence to antenatal care guidelines: secondary analysis of 2011 Ethiopian demographic and health survey data

**DOI:** 10.1186/s12884-018-1775-4

**Published:** 2018-05-09

**Authors:** Faye Forbes, Karen Wynter, Catherine Wade, Berihun M. Zeleke, Jane Fisher

**Affiliations:** 1 Jean Hailes Research Unit, School of Public Health and Preventive Medicine, Monash University, 553 St Kilda Rd, Melbourne, VIC 3004 Australia; 2 Parenting Research Centre, 232 Victoria Parade, East Melbourne, VIC 3002 Australia; 3 School of Public Health and Preventive Medicine, Monash University, 553 St Kilda Rd, Melbourne, VIC 3004 Australia; 4 Institute of Public Health, College of Medicine and Health Sciences, University of Gondar, Gondar, Ethiopia

**Keywords:** Male involvement, Male attendance, Fathers, Ethiopia, Focused antenatal care, Demographic and health survey

## Abstract

**Background:**

Complications during pregnancy, childbirth and the postpartum period present a significant and complex public health problem in low income countries such as Ethiopia. One strategy endorsed by the World Health Organisation (WHO) to improve maternal and child health outcomes is to encourage male partner involvement in pregnancy care. This research aimed to explore the relationships between 1) male attendance at antenatal care and 2) socio-economic and women’s empowerment factors and adherence to focused antenatal care guidelines among women receiving care in Ethiopia.

**Methods:**

Secondary analysis of 2011 Ethiopian Demographic and Health Survey (DHS) data. A sub-sample of couples with a child aged 0–2 years old, for whom women attended at least one antenatal care (ANC) appointment was selected. Predictor variables on socio-economic position, demographic and women’s empowerment factors, and male attendance at antenatal care were identified. Six outcome variables were constructed to indicate whether or not women: commenced ANC in the first trimester, attended at least four ANC appointments, received a urine test, received a blood test, were counselled on potential complications during pregnancy and met these focused antenatal care guidelines. Binary logistic regression was performed to estimate the relationship between the predictor and outcome variables.

**Results:**

After controlling for other factors, women whose partners attended ANC were significantly more likely to receive urine and blood tests and be counselled about pregnancy complications compared to women who attended alone. Male attendance was not associated with women commencing care in the first trimester or attending at least four appointments. Although more women whose male partners had attended appointments received all recommended components of ANC than those who attended alone, this association was not significant.

**Conclusions:**

The results revealed some benefits and did not detect harms from including male partners in focused antenatal care. Including men may require changes to maternal healthcare systems and training of healthcare workers, to adopt ‘father inclusive’ practices. Given the limited research in this area, large population studies including the DHS routinely carried out in Ethiopia could enhance knowledge by including more detailed indicators of male involvement in pregnancy, maternal and child healthcare and early child development.

**Electronic supplementary material:**

The online version of this article (10.1186/s12884-018-1775-4) contains supplementary material, which is available to authorized users.

## Background

Complications during pregnancy, childbirth and the postpartum period present a significant and complex public health problem in low income countries. In 2015 approximately 303,000 women died of pregnancy-related causes, with 99% of these deaths occurring in low- and middle-income countries and the highest proportion in Africa [1]. Many of these deaths are potentially avoidable by timely access to healthcare to prevent, identify and treat pregnancy-related problems.

Maternal and child health (MCH) were a focus of the Millennium Development Goals (MDG) (2000–2015), and continue to be prioritised in the Sustainable Development Goals (SDG) (2016–2030) agreed upon by the United Nations and co-operating countries [2]. The World Health Organisation (WHO) antenatal care (ANC) guidelines suggest a focused antenatal care model (FANC) involving four consultations and including screening tests and counselling, commencing in the first trimester and extending until birth. The WHO focus includes strategies to encourage women to attend healthcare during pregnancy, birth and in the post-natal period [3, 4].

One strategy “strongly recommended” by the WHO in the *WHO recommendations for health promotion interventions for maternal and newborn health 2015* (pp 17, [5]), is to support male involvement in pregnancy, childbirth and the post-birth period. Male involvement include men’s role in maternal health decision making, male attendance at antenatal care, male attitudes towards maternal healthcare and male participation in home visits from health extension workers [6–25]. Male involvement interventions evaluated by the WHO include: mass media campaigns, community-based outreach and education for men, home visits, counselling for couples, groups or men only, and workplace-based education [5].

The WHO encourages male involvement for diverse reasons, including that in many countries in which maternal and child health indicators do not meet international criteria, men have significant influence over maternal healthcare decisions [26, 27]. Engaging men in healthcare early in pregnancy is viewed as an opportunity to educate men about the importance of perinatal healthcare, and to help men support their partners effectively during pregnancy, birth preparation and in the postnatal period [28, 29]. This is also an appropriate time to address men’s sexual and reproductive health and to position men as responsible partners to women and fathers to their children [5].

However, the WHO acknowledge that the quality of evidence about male attendance at antenatal care is currently very low and any estimate of the effect is uncertain [5]. Two systematic reviews of the evidence in low and middle income countries presented findings from research conducted over diverse geographic regions and a range of maternal health outcomes [6, 7]. Study designs included cross-sectional demographic studies, pre-post intervention studies, retrospective cohort designs and two randomised controlled trials. Appraisal revealed limited high quality evidence with only 2/7 [7] and 6/14 studies [6] in each review rated as high quality.

Nevertheless, this evidence does tentatively suggest that in low and middle income countries men’s attendance at antenatal care consultations may be associated with a range of positive maternal outcomes [6, 7], including: birth preparedness [30, 31], birth in a medical facility rather than at home [32], access to a skilled birth attendant rather than unskilled support [7, 32–34], attendance at postnatal healthcare services and reduced postnatal depression [7, 30]. One of the few studies to produce evidence rated as high quality in Aguiar and Jennings’ 2015 review [6], was a randomised controlled trial (RCT) conducted in Nepal allocating women to a 3-arm trial consisting of women-only pregnancy and birth education, no education or couple pregnancy and birth education. The study found that women who attended antenatal care as part of a couple were better prepared for birth and used more postnatal care than women allocated to women-only or no education conditions [35].

On the other hand, male attendance at antenatal care consultations was not associated with an increase in antenatal care attendance in the RCT in Nepal [35] or with lower rates of miscarriage or stillbirth, use of antenatal care or birth preparedness in a RCT conducted in South Africa allocating men to three counselling sessions during the maternity period compared to a control condition [36].

As recognised by the WHO, involving men in pregnancy care could also be disadvantageous to women in some circumstances, primarily because it may reinforce the role of men as decision makers and provide opportunity for male coercion and control over women’s decisions [5, 37]. Women’s empowerment, rated as the sum of nine items including measures of social, economic and legal empowerment in the 2010–2011 Demographic and Health Surveys, was evaluated in eight sub-Saharan African countries, and compared between women whose male partners attended and did not attend antenatal healthcare visits [37]. Results were mixed with male attendance associated with increased indicator scores of women’s empowerment in Burkina Faso and Uganda, decreased likelihood of empowerment in Malawi and no differences detected in Burundi, Mozambique, Rwanda, Senegal, or Zimbabwe [37].

Ethiopia is a Sub-Saharan African country in which improving maternal and child health and increasing the use of healthcare during the perinatal period is a national priority [38]. Despite significant improvements to the healthcare system and maternal and child health indicators since the launch of the MDGs, the maternal mortality ratio in Ethiopia is substantially higher than international standards [38] with an estimated 350 maternal deaths per 100,000 live births, placing the country 31st on the rankings of maternal mortality ratios in a listing of 184 countries [39].

In Ethiopia, maternal healthcare is provided by government- and privately-funded primary health care facilities. Government facilities consist of a primary district hospital (serving 60,000–100,000 people), health centres (1 per 15,000–25,000 people) and Health Posts staffed by Health Extension Workers (HEW) (1 per 3000–5000 people). This is supplemented by non-government organisations and profit-based facilities [38]. In 2011 most women who received antenatal care accessed this in government facilities. From the total population 28.7% received care from a midwife /nurse, 5.4% from a doctor and 8.7% from a HEW. Only 19% received the recommended four visits, 10% had a skilled birth attendant and 10% gave birth in a health facility [40].

Despite the Ethiopian government’s focus on maternal health and the WHO recommendations regarding male involvement [5], it is not clear that the strategy of involving men in antenatal care has been implemented beyond family planning and prevention of parent-to-child transmission of HIV [38]. There are few data about the role of men in pre or postnatal care or in relation to birth preparedness in Ethiopia. A broad search of Ethiopian peri-natal care research which included information about male partners, revealed 15 quantitative, one mixed methods and one qualitative study, with quantitative samples ranging from 310 to 1750 participants with an average sample size of 724. The studies were diverse: conducted in a range of regions in urban or rural Ethiopia, and with male partner involvement defined in different ways - including male attitudes towards maternal healthcare, male partner education, co-habitation, male emotional support, male role in maternal healthcare decision making and male participation in discussions with home visiting HEWs [6–25]. Most studies did not focus exclusively on the role of men, instead examining factors related to maternal healthcare overall and including some information on male partner involvement.

Collectively these studies suggest that male involvement in perinatal care may be associated with positive maternal health outcomes in Ethiopia. Five quantitative studies showed that male partner attitude towards perinatal healthcare, approval of antenatal care and birth in a health facility, and knowledge of benefits of birth in a facility, were significantly associated with increased odds of receiving antenatal care and giving birth in a facility [9–11, 18, 20]. Two quantitative studies found that partners’ active involvement in home visiting programs for birth preparedness and antenatal education increased performance on birth preparedness measures and satisfaction with HEWs [8, 21]. One survey and one mixed methods study found male partner involvement in decision making was associated with increased odds of birth in a health facility [14, 16, 41]. Qualitative research identified that understanding of maternal health issues and financial status of the male partner contributed towards maternal healthcare use [24].

As yet there are no population level studies of associations between male partner attendance at antenatal care and perinatal healthcare use in Ethiopia, nor specific indicators of focused antenatal care, defined as beginning in the first trimester, comprising at least four sessions, and including the necessary screening and counselling components [4].

This research aimed to explore the relationships between 1) male attendance at antenatal care and 2) socio-economic and women’s empowerment factors and adherence to focused antenatal care guidelines among women receiving care in Ethiopia.

## Method

### Setting

This was a secondary analysis of data collected in the Ethiopian Demographic and Health Survey (EDHS) in 2011 [40]. The EDHS sampling procedure randomly selected discrete geographic areas or ‘clusters’ (groupings of households) across each region of Ethiopia. Regions included: Tigray, Afar, Amhara, Oromiya, Somali, Benishangul Gumuz, Southern Nations Nationalities and Peoples (SNNP), Gambella and Harari and two city administrations (Addis Ababa and Dire Dawa).

### Data source for the Ethiopian demographic and health survey

The EDHS surveys for women and men included the following sections: Respondent’s socio-demographic characteristics, Reproduction, Contraception, Marriage and Sexual Activity, Fertility Preferences, Employment and Gender Roles, HIV/AIDS and Other Health Issues. Women received additional sections: Pregnancy and Postnatal Care, Child Immunization, Health and Nutrition.

### Procedure for the Ethiopian demographic and health survey

Within each cluster 20–25 households were selected randomly from a complete list. Each household was invited to participate in a survey. Women and men between the ages of 15–49 years were invited to participate in separate surveys. The 30–60 min structured surveys comprised with fixed response options translated into Amharic, Tigrigna or Oromiffa. Interviews were conducted in the home by trained interviewers.

### Data linking and selection

**Fig. 1:**
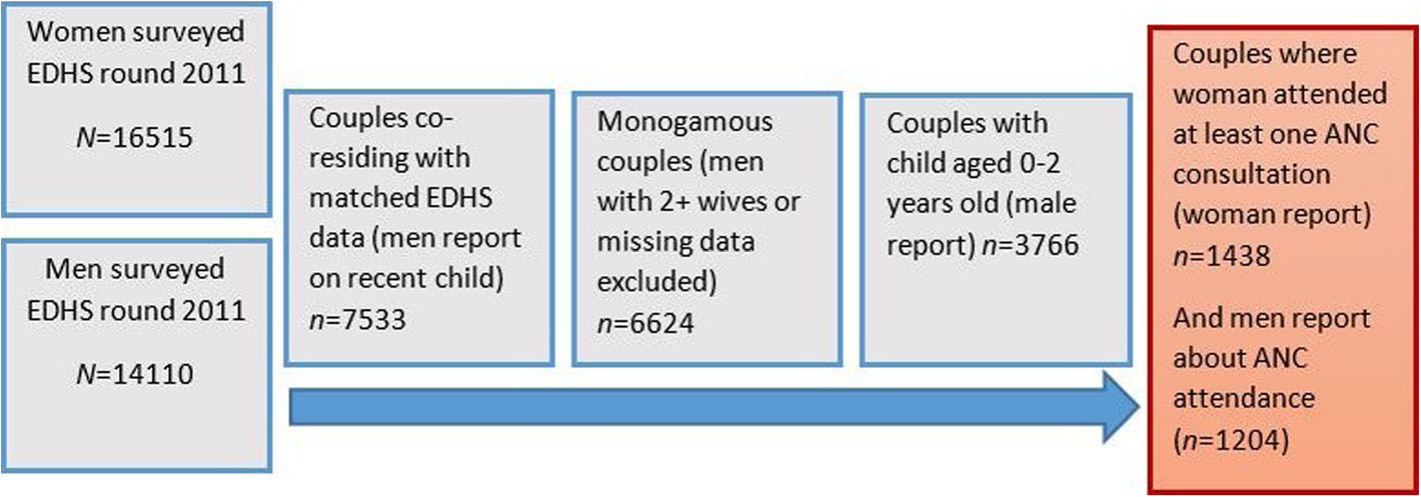
Flow chart of study data selection criteria

Data from men were linked to data from the woman they indicated was their “first wife” using a combination of the identification numbers for the household and the first wife. Data from polygamous couples were included in the DHS survey, but excluded from the analysis as the husband’s most recent child may not be the first wife’s most recent child (see Fig. 1). Data were selected for analysis if: the man responded to the question “How old is your youngest child?” by indicating that his youngest child was aged up to 2 years; if the woman responded “yes” to the question “Did you receive antenatal care for your youngest child?”; and if the man responded “Yes” or “No” to the question “Were you ever present for any of those check-ups?” (refers to antenatal care check-ups). Antenatal care was defined in the EDHS survey as “healthcare accessed during pregnancy that directly related to the woman’s pregnancy”.

### Data management and analysis

#### Key variables

The study outcome and key predictor variables, related EDHS survey items and recoded categories (where relevant) are available in a Additional file 1: Table S1.

Secondary analysis was performed on EDHS data using IBM SPSS Statistics version 23.

Descriptive statistics were used to summarise the demographic and socio-economic characteristics of study participants. Data were recoded to reduce the number of categories and aid interpretation (see Additional file 1: Table S1). Significant differences between men who did and did not attend at least one antenatal care appointment with their partner were calculated using independent sample t-tests and chi-square tests. *P*-values of *p* < 0.05 are reported as significant in all analyses.

Bivariate analysis was used to identify associations between outcome and predictor variables. Binary logistic regression was performed to estimate the relationship between the predictor variables and outcomes, reporting the Adjusted Odds Ratio (AOR) and 95% confidence interval (CI). The relationships between each predictor variable and the antenatal care outcome variables were evaluated while controlling for the effect of other variables (residence, education, age, parity, location of ANC, male partner attendance, wealth, role in MCH decision, attitude towards “wife beating” and exposure to media) contained in the model. All variables in each model were significantly associated with the relevant outcome variable and male attendance at antenatal care. Only cases where data were available for each indicator were kept in the model. Nagelkerke score was used to compare goodness of fit between models.

It is possible that male attendance at antenatal appointments may have a negative impact on antenatal care outcomes for some women, for example those in relationships characterised by coercion, control or physical and sexual violence perpetrated by an intimate partner. As a sensitivity analysis, we conducted bivariate analyses of associations between the outcome variables and male attendance separately for two groups of women, according to their response to a question “In your opinion, is a husband justified in hitting or beating his wife in the following situations: If she goes out without telling him?, If she neglects the children?, If she argues with him?, If she refuses to have sex with him?, If she burns the food?”. The latter was used as a proxy indicator for intimate partner violence. A series of 3-way cross-tabulations with chi-square tests was conducted. If significant associations between an outcome variable and male attendance exist for one group, according to response to this question, and not for the other group, then a significant interaction between male attendance and presence of intimate partner violence would be indicated, and would subsequently be tested in the models.

## Results

### Sample characteristics

Complete data were available for 1204 couples who met inclusion criteria. The sample characteristics are presented in Table 1. Compared to men who did not attend, men who attended at least one antenatal care appointment with their partner were younger, had fewer children, were more educated, were wealthier, were more likely to live in an urban area, report partner or shared healthcare decision making, follow Ethiopian Orthodox religion and have exposure to media and were less likely to have a partner who reported, or to report themselves, that “wife beating” was acceptable in any circumstances.

**Table 1:** Characteristics of women and men in couples meeting inclusion criteria: n(%)

Characteristics n (%)	Men and women with children 0–2 with partners who attended at least one ANC session (included in study)			
	Women included in study	Men who attended ANC	Men who did not attend ANC	Sig differences between men
Age (Mean SD)	28.47 (6.72)	34 (7.37)	35.34 (8.02)	***
Number of children (Mean SD)	4.08 (2.62)	3.45 (2.74)	4.23 (2.76)	***
Education				
No school	776 (54%)	152 (27%)	228 (36%)	***
Primary	498 (35%)	263 (47%)	314 (49%)	NS
Secondary	103 (7%)	78 (14%)	57 (9%)	***
Higher	61 (4%)	69 (12%)	44 (7%)	***
Wealth Index				
Poorest quintile	217 (15%)	52 (9%)	104 (16%)	***
Poor quintile	217 (15%)	72 (13%)	97 (15%)	NS
Average quintile	241 (17%)	77 (14%)	109 (17%)	NS
Rich quintile	273 (19%)	100 (18%)	127 (20%)	NS
Richest quintile	484 (34%)	260 (46%)	206 (32%)	***
Residence				
Addis Ababa	121 (8%)	77 (14%)	44 (7%)	***
Other urban	308 (21%)	154 (27%)	136 (21%)	***
Rural	1009 (70%)	330 (59%)	463 (72%)	***
Healthcare decisions				
Woman alone	226 (16%)	108 (19%)	87 (14%)	*
Shared	939 (65%)	356 (64%)	445 (69%)	*
Man alone	271 (19%)	96 (17%)	110 (17%)	NS
Religion				
Orthodox	613 (43%)	276 (49%)	279 (43%)	***
Catholic	10 (1%)	3 (0%)	6 (1%)	NS
Protestant	256 (18%)	78 (14%)	106 (16%)	NS
Muslim	548 (38%)	199 (35%)	245 (38%)	NS
Traditional	9 (1%)	1 (0%)	1 (0%)	
Media exposure				
At least once per week	495 (34%)	416 (74%)	385 (60%)	***
Does not have access to TV, radio or newspaper	943 (66%)	145 (26%)	258 (40%)	***
Attitudes towards “wife beating” (women)				
Acceptable in at least one condition	944 (66%)	333 (59%)	436 (68%)	**
Never acceptable	494 (34%)	228 (41%)	207 (32%)	**

*** sig at *p* < .001 ** sig at *p* < .01 * sig at *p* < .05

Components of focused antenatal care compared on the basis of whether a woman’s partner did or did not attend at least one antenatal care appointment are presented in Table 2. Women whose partner attended at least one antenatal care session were more likely to attend antenatal care in a private facility, to commence care in the first trimester of pregnancy, to attend four or more antenatal care sessions, to complete urine and blood screening tests during antenatal care and to be counselled about pregnancy complications than those who attended without a partner.

**Table 2:** Antenatal care variables for women whose partners did and did not attend antenatal care: n (%)

Antenatal care and pregnancy variables	Men who attended ANC *n* = 561	Men who did not attend ANC *n* = 643	Sig
Place woman received ANC			
Government facility	460 (84%)	595 (94%)	***
Private facility	78 (14%)	28 (5%)	***
Missing	15 (3%)	14 (2%)	NS
Trimester during which woman commenced antenatal care			
First trimester	196(35%)	181 (29%)	*
Second trimester	293(53%)	362 (57%)	NS
Third trimester	68 (12%)	93 (15%)	NS
Number of ANC consultations for woman			
< 4 ANC sessions	258 (46%)	340 (53%)	**
≥ 4 ANC sessions	300 (54%)	299 (47%)	**
Missing	3 (0%)	4 (1%)	
Screening tests during ANC woman			
Blood test	405 (72%)	380 (59%)	***
Urine test	331 (59%)	270 (42%)	***
Counselled about potential pregnancy complications			
Not told complications	394 (70%)	523 (82%)	***
Told complications	167 (30%)	119 (19%)	***

*** sig at *p* < .001 ** sig at *p* < .01 * sig at *p* < .05

As described in the method section, associations between male attendance at antenatal care and outcome measures were compared between women who did and did not report that “wife beating” was acceptable. There were no differences.

### Logistic regression analyses

#### Commencing antenatal care in the first trimester

Women in households classified by the DHS as being in the poorest, poor and middle wealth quintiles were approximately half as likely to begin care in the first trimester compared to those in households classified as being in the richest wealth quintile. Women receiving care in a government facility were less likely to commence care in the first trimester than those attending a private facility. After controlling for socio-economic and demographic factors, there was no significant association between male attendance at antenatal care and commencing ANC in the first trimester (see Table 3).

**Table 3:** Multivariate logistic regression results for each outcome measure: Adjusted Odds Ratios (AOR) (95% Confidence Interval)

	First trimester (*n* = 1162)	Attended 4+ ANC (*n* = 1166)	Urine sample ANC (*n* = 1172)	Blood sample ANC (*n* = 1173)	Counselling ANC (*n* = 1168)	All components ANC (*n* = 1173)
Addis Ababa (reference)						
Urban	1.14(.71–1.83)	***.17(.08–.34)	***.07(.02–.31)	*.08(.01–.61)	.64(.40–1.02)	.68(.38–1.24)
Rural	.77(.42–1.40)	***.17(.08–.37)	***.03(.01–.13)	**.03(.00–.25)	*.57(.28–.96)	*.28(.09–.84)
Higher Education (reference)						
No Education	.84(.48–1.45)	***.32(.17–.59)	.64(.33–1.22)	1.08(.55–2.14)	*.57(.33–1.00)	.46(.18–1.17)
Primary Education	.79(.49–1.28)	***.35(.20–.61)	.57(.31–1.05)	.97(.51–1.86)	*.57(.36–.93)	**.34(.17–.65)
Secondary Education	1.32(.76–2.31)	*.49(.25–.93)	.91(.44–1.90)	1.31(.59–2.90)	.68(.39–1.17)	.75(.39–1.45)
Mother Age	1.03(.99–1.06)	**1.05(1.02–1.09)	.20(.96–1.04)	*1.04(1.0–1.08)	1.01(.97–1.05)	1.01(.95–1.07)
Number of Children	.91(.82–1.00)	**.87(.78–.94)	.92(.84–1.02)	.92(.84–1.02)	.98(.89–1.08)	.89(.73–1.08)
Private clinic ANC (reference)						
Public Health Service ANC	*.36(.23–.57)	.61(.37–1.01)	*.48(.26–.86)	1.21(.67–2.18)	.82(.52–1.31)	*.50(.28–.91)
Male Attendance (reference)						
No Male Attendance	1.05(.79–1.39)	1.06(.82–1.38)	*.73(.55–.97)	*.70(.53–.93)	**.64(.48–.86)	.65(.39–1.10)
Richest Wealth quintile (reference)						
Poorest Wealth quintile	**.43(.23–.80)	**.37(.21–.65)	***.34(.19–.60)	***.35(.20–.62)	.61(.32–1.19)	.20(.02–1.80)
Poor Wealth quintile	*.51(.28–.93)	.61(.35–1.04)	**.46(.26–.81)	**.42(.24–.74)	.60(.31–1.16)	.40(.07–2.24)
Middle Wealth quintile	*.54(.30–.97)	.62(.37–1.05)	**.41(.23–.70)	***.36(.21–.64)	*.50(.26–.96)	.36(.06–2.00)
Rich Wealth quintile	.61(.36–1.03)	.76(.47–1.24)	.66(.41–1.08)	**.49(.29–.82)	.69(.39–1.21)	.56(.16–1.94)
MCH Decision Woman (reference)						
MCH Decision Shared	.82(.56–1.18)	.97(.67–1.42)	.74(.49–1.11)	.80(.52–1.22)	1.18(.80–1.74)	1.21(.67–2.20)
MCH Decision Man	.86(.59–1.08)	.82(.60–1.30)	.71(.43–1.07)	.96(.58–1.58)	1.03(.61–1.72)	1.19(.48–2.94)
“Wife beating” not acceptable (reference)						
“Wife beating” acceptable	.80(.60–1.09)	.86(.64–1.14)	*.68(.50–.93)	*.69(.50–.94)	.76(.55–1.04)	.93(.55–1.59)
Media Weekly (reference)						
Media Less than weekly	.96(.68–1.35)	.91(.68–1.22)	.83(.61–1.14)	.81(.60–1.09)	.88(.60–1.28)	.43(.14–1.33)
Nagelkerke R2	.182	.229	.398	.284	.132	.330

*** sig at *p* < .001 ** sig at *p* < .01 * sig at *p* < .05

### Number of antenatal visits

Women were significantly less likely to attend at least four ANC visits if they lived in urban centres and rural areas compared to the capital city, and if their partner had no education, primary or secondary education compared to higher education. The likelihood of receiving four ANC sessions increased as maternal age increased, but decreased as the number of children increased. Women in the poorest quintile were significantly less likely to commence care in the first trimester compared to those in the wealthiest quintile. After controlling for socio-economic and demographic factors, there was no significant association between male attendance at ANC and receiving the recommended number of visits (see Table 3).

### Urine sample and blood sample collected during ANC

Women attending antenatal care without a male partner were significantly less likely to receive both a urine and a blood test compared to those whose partners attended (Table 3). In addition, women were significantly less likely to provide both kinds of samples for screening tests if they lived in other urban or rural areas than those in the capital, and if they were in poorer wealth quintiles compared to the richest wealth quintile. Women who said “wife beating” was acceptable in some circumstances were significantly less likely to receive both kinds of tests, compared to those who said violence was never acceptable.

Women were less likely to report a urine sample if they attended a government facility for ANC compared to those receiving ANC at a private clinic. Women were more likely with increasing age to receive a blood test (see Table 3).

### Counselled about pregnancy warning signs during antenatal care

Women attending antenatal care without a male partner were significantly less likely to report that they had been counselled about possible complications during pregnancy (Table 3). Women were significantly less likely to report being warned of complications if they lived in rural areas compared to those in Addis Ababa, if they were in the middle wealth quintile compared to the richest and if their partners had no education or only primary education compared to those with higher education (see Table 3).

### All recommended components of focused antenatal care

Women were significantly less likely to receive all recommended components if they lived in rural areas compared to the capital city, and if their partner had only primary education compared to higher education. Women were also significantly less likely to receive all components if they attended a public health facility compared to a private facility. After controlling for socio-economic and demographic factors, there was no significant association between male attendance at ANC and receiving all recommended components of ANC (see Table 3).

## Discussion

This is the first study examining male attendance at antenatal care and factors associated with meeting WHO-recommended components of antenatal care in Ethiopia, a country with low rates of healthcare utilisation, high rates of maternal mortality and high rates of shared decision making [5, 40]. Strengths include the country-wide randomly selected sample of women already accessing care. It builds upon existing research which has focused mainly on Ethiopian women’s involvement in *any* antenatal care [23–25], and examines for the first time, whether male attendance at antenatal care visits is associated with enhanced antenatal care, which includes screening tests, counselling and recommended timing of and amount of antenatal care. The data are from the first round of EDHS surveys to ask men about attendance at antenatal care [6]. Moreover an investigation of male attendance at antenatal care as a potential strategy for improving maternal and child health outcomes in Ethiopia has not previously been reported in published systematic reviews on the topic, [6, 7].

### Study limitations

Alongside the benefits of using rigorously collected population level data, there are limitations to secondary data analysis, specifically the study was limited to questions regarding male involvement already included in the Ethiopian Demographic and Health Survey. Further, the sample is representative of women accessing antenatal care, but does not allow generalisations to the entire population of women with children in Ethiopia many of whom do not access antenatal care.

### Factors associated with comprehensive antenatal care

Consistent with previous research [14], these data suggest commencing care in the second or third trimester of pregnancy is common: only 32% of women included in the study had begun antenatal care in the first trimester (Table 3). Late presentation may be common across sectors of society because of lack of family planning, access to care or because of traditional views on healthcare during pregnancy, irrespective of urban or rural location, education, mother’s age, empowerment, exposure to media or number of children. Wealth was the only significant factor in seeking care early – with women in the highest wealth quintile beginning before all other wealth groups. It is possible that the costs associated with healthcare (which may include lengthy transport) are prohibitive, or couples belonging to the wealthiest sub-cultures are more likely to plan for pregnancy, and thus detect the early signs and access antenatal care.

The study results are consistent with previous research across many low income countries, which indicate healthcare utilisation is positively associated with socio-economic variables including wealth and education [42]. The findings are also similar to results of international research in which women were found to be less likely to attend antenatal care as they grow older and have more children [35]. Possibly this is associated with the growing demands of caring for an increasing number of children, resulting in difficulty attending healthcare, or growing familiarity with pregnancy and childbirth.

The most significant factor associated with attending the recommended amount of antenatal care, receiving screening tests and receiving all the components of antenatal care was where the woman lived. Living in the capital city significantly increased a woman’s chance of receiving four antenatal care sessions and undergoing screening tests, independent of the influence of wealth, education or any other factor. Previous research using EDHS data has examined place of residence using an urban/rural dichotomy without presenting information on whether urban centres are different to the capital city [42, 43]. Our findings indicate the need for caution in interpreting urban antenatal statistics for Ethiopia, which may reflect indicators of health care in Addis Ababa (and possibly some regional capital cities), but not other urban centres. Residing in the capital city may be associated with system level factors (for example better equipped facilities and more accessible health centres) and individual level factors (for example more education about the importance of family planning and accessing healthcare during pregnancy) that facilitate meeting guidelines for focused antenatal care.

Variables which may indicate women’s empowerment (participation in decision making and access to media) did not make a significant, independent contribution to any of the antenatal care outcomes when other factors were controlled. This is inconsistent with previous international research that found women’s decision making and autonomy significantly impacted the likelihood of them accessing healthcare [27]. In previous studies, a greater proportion of women did not participate in healthcare decisions at all; thus the current study only included women receiving antenatal care and who had greater rates of participation in their own healthcare decisions – and may be considered more empowered. Which highlights the need for caution in generalising these results to the broader Ethiopian population.

Finally, after adjusting for socio-economic position, attending antenatal care from a private provider compared to the public health system was one of the few indicators that significantly increased a women’s chance of receiving all recommended components of antenatal care. This might indicate that facility-related factors (such as equipment, training and caseload) and individual factors (such as attitude towards healthcare) are combined in women attending private clinics, and lead to these women being more likely to receive all components of antenatal care.

### Associations with male attendance at antenatal care

Consistent with a growing body of international and Ethiopian research describing enhanced maternal health outcomes associated with male involvement in maternal healthcare [6–25], higher rates of screening tests (urine and blood samples) and counselling about potential pregnancy complications were significantly associated with male partner attendance at antenatal care. These associations remained significant after controlling for socio-economic factors, women’s decision making power and location of antenatal care.

Men’s attendance, combined with the fact that resources such as time and money have been made available, may indicate men’s approval for care, which is associated with increased antenatal care use [9–11]. An alternative mechanism for the association between male attendance and antenatal care outcomes could be that male approval for care and understanding of the benefits of care led the couple to select better equipped facilities with better trained staff. Having a male partner present may also have elicited more comprehensive levels of care from the same facility, because the couple together were more capable of paying on the spot fees for screening tests, or able to ask more questions and elicit more in-depth counselling from health workers. Alternatively, health care provider behaviour may be different if a male partner is present. Due to cultural status of men as the head of a family, health workers may have been more likely to offer the necessary screening and counselling tests for couples than for women alone. On the other hand, differences in screening tests and counselling could also be related to factors unaccounted for in the model. For example, partner attendance could signal his anxiety about the pregnancy rather than approval of health services, which in turn prompts selecting the most equipped facility, the most skilled providers or all the necessary tests.

After controlling for socio-economic and demographic factors, women attending alone were only 35% as likely to receive all the necessary components of antenatal care compared to women who attended with a male partner. However in this sample the association was not statistically significant. Nevertheless the substantial AOR lends support to the argument there are benefits in antenatal care associated with male attendance in Ethiopia.

Contrary to previous international and Ethiopian research [9–11, 35] and different to the other indicators of best-practice antenatal care in this study, after controlling for socio-economic factors, neither commencing care in the first trimester nor attending at least four appointments was significantly associated between male attendance. The discrepancy with previous Ethiopian research is likely to be methodological, specifically due to differences between the samples of women in the studies along with differences in the criteria for defining antenatal care use. Previous research in Ethiopia used samples of women selected because of a recent birth, irrespective of whether they accessed any care [9–11], whereas the current study was limited to women who received at least one consultation. Other studies have defined antenatal care utilisation as either a binary variable (attending or not), or as a continuous variable commencing at none, whereas the current study evaluated commencing care in the first trimester or attending at least four sessions. The definitions that include the option of no care appear more sensitive to male partner involvement.

Research from India and Nepal found male participation in antenatal care was associated with improvements in the use of skilled birth attendants and postnatal care use following targeted interventions [33, 35]. The current study did not incorporate consideration of whether the couple received systematically targeted information on any aspect of perinatal care. The results may suggest that male attendance at antenatal care without a targeted intervention beyond usual care is not associated with commencing care early or attending four sessions in women already attending care in Ethiopia.

### Implications for practice, programmes, policy and future research

Including men in antenatal care is likely to entail changes to maternal healthcare systems and healthcare worker training, to adopt more ‘father inclusive’ practices, and as recommended by the WHO [5], may need specific attention to ensure women’s autonomy is not compromised by male inclusion. Routine measurement of adherence to antenatal care protocols is also advised, regardless of whether women attend alone or with a male partner.

The results indicate that health promotion strategies relating to antenatal care attendance are best targeted outside the capital, focusing on other urban centres and rural areas to reach underserved populations. Given the importance of pregnancy complication warnings to large numbers of women giving birth at home, particular focus is needed on improving the counselling aspect of antenatal care for all women and their partners across Ethiopia. Focused antenatal care (FANC) guidelines emphasise the importance of counselling for women, and the FANC Ethiopian training module stresses a “shared responsibility for complication readiness and birth preparedness”, stating that FANC should “alert the family in all pregnancies for potential complications which could occur at anytime” [4]. The finding that only a small proportion of women report being counselled on pregnancy complications is inconsistent with FANC guidelines and suggests antenatal care implementation has deviated from Ethiopian guidelines and WHO recommendations [4, 5].

Based on previous research male involvement may increase the likelihood of women attending ANC. This study examined whether it was associated with making women’s care comprehensive enough to comply with WHO guidelines. As there were no associations between commencing care early or attending the minimum recommended number of ANC visits, caution should be applied in relying completely on male-focused strategies to encourage recommended antenatal care attendance.

Finally, future research on male involvement in antenatal care in Ethiopia is needed in order to understand women’s, men’s and health care workers attitudes towards male involvement to better understand the cultural context, potential harms, potential benefits, the barriers and the facilitators to accommodate changes in what is traditionally considered a woman’s domain. In addition, there is need for controlled evaluation of targeted health promotion strategies aimed at men to examine the influence of specifically providing men with education on perinatal care. In addition, large population studies routinely carried out in Ethiopia could greatly enhance knowledge in this area if they included more detailed indicators of male involvement in pregnancy, maternal and child healthcare and early child development. Male involvement during pregnancy may have a range of additional benefits to maternal and child health, including post-natal care use, skilled birth attendance, improved partner support, maternal mental health and increased bonding between father and child [36]. Alternatively, there could be harms from male involvement not captured by existing research. Consequently, there is a need for greater exploration of the nature and role of male involvement in maternal and child health more broadly in Ethiopia.

## Conclusions

In conclusion, consistent with WHO recommendations and previous research, the findings provide evidence that male attendance at antenatal care in Ethiopia may be associated with women receiving more comprehensive antenatal care. On balance the results revealed some benefits and did not detect harms from including male partners in antenatal care in Ethiopia. This is relevant to policy makers, practitioners and researchers working in public health in Ethiopia.

## Additional file

Additional file 1: pdf contains a supplementary table listing original survey items, response categories and coding for analysis. (PDF 367 kb)

## Abbreviations

ANC: Antenatal care
AOR: Adjusted odds ratio
DHS: Ethiopian demographic and health survey
EDHS: Ethiopian demographic and health survey
FANC: Focused antenatal care
HEW: Health extension workers
IRB: Institutional review board
MCH: Maternal and child health
MDG: Millennium development goals
RCT: Randomised controlled trial
SDG: Sustainable development goals
SNNP: Southern Nations nationalities and peoples
WHO: World health organisation
