# Supplementary material for: Male partner attendance at antenatal care and adherence to antenatal care guidelines: secondary analysis of 2011 Ethiopian demographic and health survey data
Source: BMC Pregnancy Childbirth. 2018 May 9;18:145. doi: 10.1186/s12884-018-1775-4 (PMC5944090; doi:10.1186/s12884-018-1775-4)
Supplement: Supplementary file 1 — pdf contains a supplementary table listing original survey items, response categories and coding for analysis. (PDF 367 kb) [file 12884_2018_1775_MOESM1_ESM.pdf]

Supplementary Table. Outcome and predictor variables in relation to the EDHS survey items

Study variables and EDHS Survey Questions	***EDHS response categories***	***Recoded response categories, where relevant***
**Woman's questionnaire**		
*Trimester began ANC**		
"How many months pregnant were you when you first received antenatal care for this pregnancy?"	Number/ Don't know	1=began in 1st trimester, 0=began in 2nd or 3rd trimester
*Number of ANC visits**		
"How many times did you receive antenatal care for this pregnancy?"	Number/ Don't know	1=attended 4+ ANC visits, 0=attended <4 ANC visits
Location of ANC visits		
"Where did you receive antenatal care for this pregnancy?"	Your home, other home/ Government hospital, health centre, health post, other/ Private hospital, clinic, other	1=government facility<br>2=private facility
*Urine sample, Blood sample**		
"As a part of your care this pregnancy were any of the following done at least once: Did you give a urine sample? Did you give a blood sample?"	Y/N each option<br>Don't know	NA
*Told of complications**		
"During (any of) your antenatal care visit(s), were you told about things to look out for that might suggest problems with the pregnancy?"	Y/N/<br>Don't know	NA
Mother age		
"How old were you at your last birthday?"	Age in years	NA
Woman decision power		
"Who usually makes decisions about health care for yourself: you, your (husband/partner), you and your (husband/partner) jointly, or someone else?"	Woman/ Partner/ Couple/ Someone else/ Other	1=woman<br>2=woman & partner<br>3=partner
Region		
Sampling domain determined by administration boundaries: Tigray, Afar, Amhara, Oromiya, Somali, Benishangul Gumuz, Southern Nations Nationalities and Peoples (SNNP), Gambella, Harari, Addis Ababa and Dire Dawa. Within each region participants allocated to either urban or rural status.		1=Addis Ababa, 2=Other urban centre, 3=Rural
Number of children		
Calculated by adding entries in mother's birth history (including children who have died after birth)		NA
Wealth index		
Constructed from household assets and characteristics (42)		NA
Attitudes to wife beating		
In your opinion, is a husband justified in hitting or beating his wife in the following situations:<br>If she goes out without telling him?	Y/N/<br>Don't know	1=Violence acceptable in at least one condition

	If she neglects the children?<br>If she argues with him?<br>If she refuses to have sex with him?<br>If she burns the food?		0=Violence not acceptable in any condition
**Man's questionnaire**			
Male attendance at antenatal care			
	(If men indicated their partner received antenatal care for most recent child) Were you ever present for any of those check-ups?	Y/N/<br>Don't know	NA
Education			
	"What is the highest level of school you attended?"	None/Primary/Secondary/ Higher	NA
Exposure to media			
	"Do you read a newspaper/magazine?"<br>"Do you watch TV?"<br>"Do you listen to the radio?"	At least once p/wk/<br>Less than once p/wk/<br>Not at all	1=At least once per week TV or radio or newspaper, 0=less than once per week for TV and radio and newspaper

*Outcome variable
